# From cellular to molecular mechanobiology

**DOI:** 10.1063/1.5129937

**Published:** 2020-02-14

**Authors:** Cheng Zhu, Cho-yin Lee, Larry V. McIntire

**Affiliations:** 1 Wallace H. Coulter Department of Biomedical Engineering, Georgia Institute of Technology, Atlanta, Georgia 30332, USA; 2 George W. Woodruff School of Mechanical Engineering, Georgia Institute of Technology, Atlanta, Georgia 30332, USA; 3 Parker H. Petit Institute for Bioengineering and Bioscience, Georgia Institute of Technology, Atlanta, Georgia 30332, USA; 4 Department of Biomedical Engineering, National Yang-Ming University, Taipei 11221, Taiwan; 5 Department of Radiation Oncology, Taoyuan General Hospital, Ministry of Health and Welfare, Taoyuan 33004, Taiwan

## Abstract

Mechanobiology at the cellular level is concerned with what phenotypes that cells exhibit to maintain homeostasis in their normal physiological mechanical environment, as well as what phenotypical changes that cells have to make when their environment is altered. Mechanobiology at the molecular level aims to understand the molecular underpinning of how cells sense, respond to, and adapt to mechanical cues in their environment. In this Perspective, we use our work inspired by and in collaboration with Professor Shu Chien as an example with which we connect the mechanobiology between the cellular and molecular levels. We discuss how physical forces acting on intracellular proteins may impact protein–protein interaction, change protein conformation, crosstalk with biochemical signaling molecules, induce mechanotransduction, and alter the cell structure and function.

## INTRODUCTION

As the inner lining of the blood vessel wall, the endothelium experiences a myriad of forces exerted by the circulating blood that vary in space and time. Mechanobiology of the vascular endothelium studies how endothelial cells sense, respond to, and adapt to their complex mechanical environment, aiming to understand how endothelial cells maintain homeostasis in their normal physiological mechanical environment, as well as how they adapt when such a mechanical environment is altered.^1^ Failure to adapt results in endothelial cell dysfunction and diseased blood vessels, manifested as the formation of arteriosclerotic plagues and other abnormalities, potentially leading to cardiovascular diseases. Top on the list of such diseases are heart attack and stroke, which are major causes of mortality and morbidity in the United States, Europe, and now also China.^2^ Thus, vascular endothelium mechanobiology represents a point of intersection between mechanobiology and vascular biology and physiology. Simplistically speaking, it may be broken down into tissue, cellular, and molecular levels according to the length scales. This Perspective discusses some of the studies that bridge the latter two levels, including, among other topics, the endothelial cells' response to shear stress and cyclic pressure of the blood flow, including the mechanosensing molecules and mechanisms, intracellular signaling pathways, up- and down regulation of mechanosensitive genes, expression of and regulation by microRNAs, and the altered cellular functions.

Much of the research in the field follows a paradigm that mechanosensing begins at the surface of the endothelial cell through membrane structures, including membrane proteins, lipids, and glycocalyx. Mechanotransduction across the cell membrane launches a cascade of chemical reactions, including protein–protein interactions and enzymatic modifications, which amplify and integrate the signals coming from various sources to arrive at a decision. This decision is then relayed to the nucleus to change the gene expression program and, in turn, the cell's properties and function. Many papers have been published on the above topics, and the readers are referred to the excellent reviews by authorities of the field,^3–5^ including some written by Professor Shu Chien^1^ and by one of us.^6,7^

Although much less has been done, recent developments in the field have included exciting studies on the mechanotransduction of intracellular proteins, including adaptor proteins in the focal adhesion complex, cytoskeletal proteins, and nuclear proteins. Using as an example a joint project between the Cheng Zhu and Larry McIntire laboratories inspired by Professor Shu Chien's work and performed by Cho-yin Lee, we review some of these recent studies and suggest promising areas for future studies.

## CONCEPTUALIZATION AND RATIONALE

**FIG. 1.**
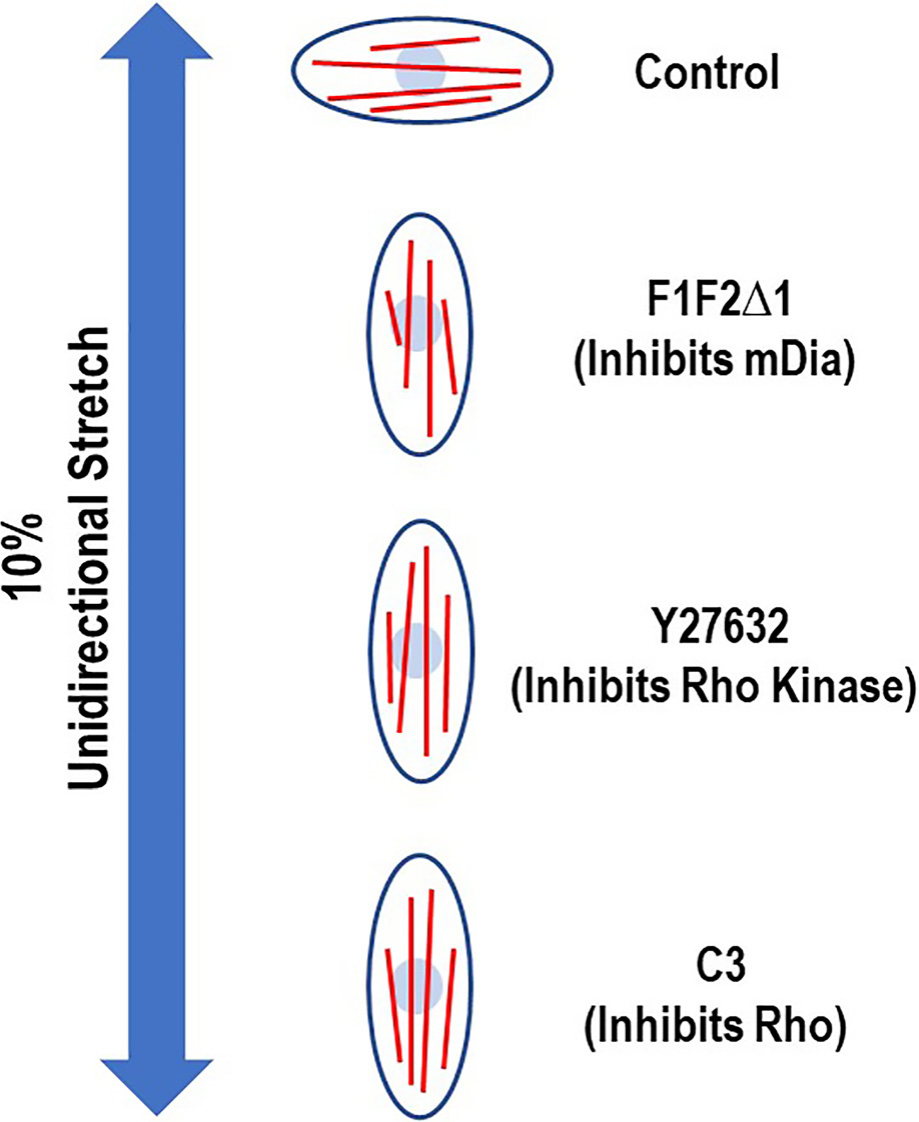
Schematic illustration of the effect of Rho, Rho kinase, and mDia on force-induced actin stress fiber organization. In the study by Kaunas *et al.*,^8^ a cyclic unidirectional stretch (10%, 1 Hz) was applied to bovine aortic endothelial cells cultured on the elastic membrane. The actin stress fibers (red lines) were induced mechanically to form and align perpendicular to the stretch direction (control). When cells were transfected by F1F2Δ1, which inhibits mDia, or treated by Y27623, which inhibits Rho Kinase, or C3, which inhibits Rho, the stress fibers were switched to align in parallel to the stretch direction. Sketched to depict the data from Ref. 8.

In a 2006 article, the Chien lab demonstrated that force regulates the alignment of intracellular actin stress fibers, which is modulated by molecules of the Rho pathway.^8^ In the study, a cyclic uniaxial strain was applied to the elastic membrane on which bovine aortic endothelial cells were cultured. The applied strain mimics the hoop strain in the arterial wall caused by pulsatile blood pressure due to heart beats. The authors observed an augmented formation of intracellular actin stress fibers, which was aligned perpendicular to the strain direction. Interestingly, this alignment switched direction and became parallel to the strain direction when Rho and its downstream effectors, Rho kinase and mDia, were inhibited (Fig. 1).

In his 2016 Cannon Award Lecture review article entitled “Mechanotransduction and endothelial cell homeoastasis: the wisdom of the cell,”^1^ Professor Shu Chien summarized his work on mechanotransduction with a unified concept: endothelial cells can adapt to mechanical environments with a regular directional pattern (e.g., sustained directional shear stress and stretch, respectively, caused by blood flow and pressure at the straight parts of the arterial tree) to result in an atheroprotective phenotype, but not mechanical environments without a directional pattern (e.g., irregular shear stresses and stretches at arterial branches with complex geometries), which induce pro-inflammation and proliferative signals to result in an athero-prone phenotype. According to this view, the observed alignment of intracellular actin stress fibers under cyclic uniaxial stretch can be regarded as the cells' homeostatic phenotype. The re-orientation of the alignment may reflect an effort to adapt to a changed mechanical environment sensed by the cells. The Rho-formin module may represent key signaling molecules regulating such mechanosensing and adaptation.

Intrigued by the above observations and their potential functional significance in mechanosensing and adaptation of cells, Lee wanted to explore the molecular mechanism underpinning this phenomenon in his Ph.D. thesis research as a joint project between the laboratories of Cheng Zhu and Larry McIntire. At that time, the Zhu lab had just demonstrated catch bonds in the interactions of P-selectin^9^ and L-selectin^10^ with their common and distinct ligands. Catch bonds are an unusual phenotype of dynamic molecular interaction (dynamic bonds) where force counter-intuitively prolongs bond lifetime, which are in contrast to the ordinary slip bond phenotype where force shortens bond lifetime.^11^ Experiments were under way to determine whether platelet glycoprotein Ib (GPIb) and integrins formed catch bonds with their respective ligands, which was soon shown to be the case for GPIb–von Willebrand factor (VWF),^12^ α_5_β_1_–fibronectin,^13^ and α_L_β_2_–intercellular adhesion molecule-1 (ICAM-1)^14^ interactions. Therefore, we sought to employ single-bond mechanical techniques to investigate the underlying mechanism of the Rho pathway regulated force modulation of actin stress fiber alignment.

To articulate the rationale, we noted that, just like the above extracellular receptor–ligand bonds that often have to support applied forces, intracellular molecules may also be subjected to endogenous forces. As a general premise, interactions among force-generating and/or force-bearing molecules may be modulated by force.^15^ In particular, actin stress fibers bear force generated by myosin motors or by actin polymerization itself, or transmitted from extracellular sources via adhesion molecules.^16–19^ As such, force must modulate the dynamic reorganization of the actin cytoskeleton in cells, potentially through the modulation of the turnover of the polymeric filamentous actin (F-actin) assembled from monomeric globular actin (G-actin) by non-covalent interactions.^18–20^ This rationale led us to test the first hypothesis of the project: Tensile force modulates the dissociation kinetics of actin subunits.

To connect such a biophysical hypothesis to the biological mechanism underlying the regulation of force induction of actin stress fiber alignment by molecules of the Rho pathway, we noted that actin polymerization and depolymerization are also regulated biochemically by actin binding proteins. Among them, mDia, which was shown by Kaunas *et al.* to regulate force-induced (re)alignment of actin stress fibers in cells,^8^ is a subclass of Rho-GTPase effector formins (formin homology proteins). The formin active domain FH2 accelerates actin nucleation and stabilizes F-actin barded ends through direct binding to actin subunits.^21–26^ The mammalian formin mDia1 is a representative of mDia isoforms and is controlled by RhoA via an auto-inhibition module. The FH2 domain of mDia1 is auto-inhibited by the interaction between the N-terminal diaphanous inhibitory domain (DID) and the C-terminal diaphanous auto-regulatory domain (DAD) and binding of RhoA to mDia1 disrupts the DAD–DID interaction, thereby relieving the auto-inhibition to activate the FH2 domain.^21,22,24^ This background led us to test the second hypothesis of the project: the force modulation of actin depolymerization kinetics, if it exists, is cross-regulated by RhoA and formin.

## SINGLE ACTIN BOND LIFETIME UNDER FORCE

**FIG. 2.**
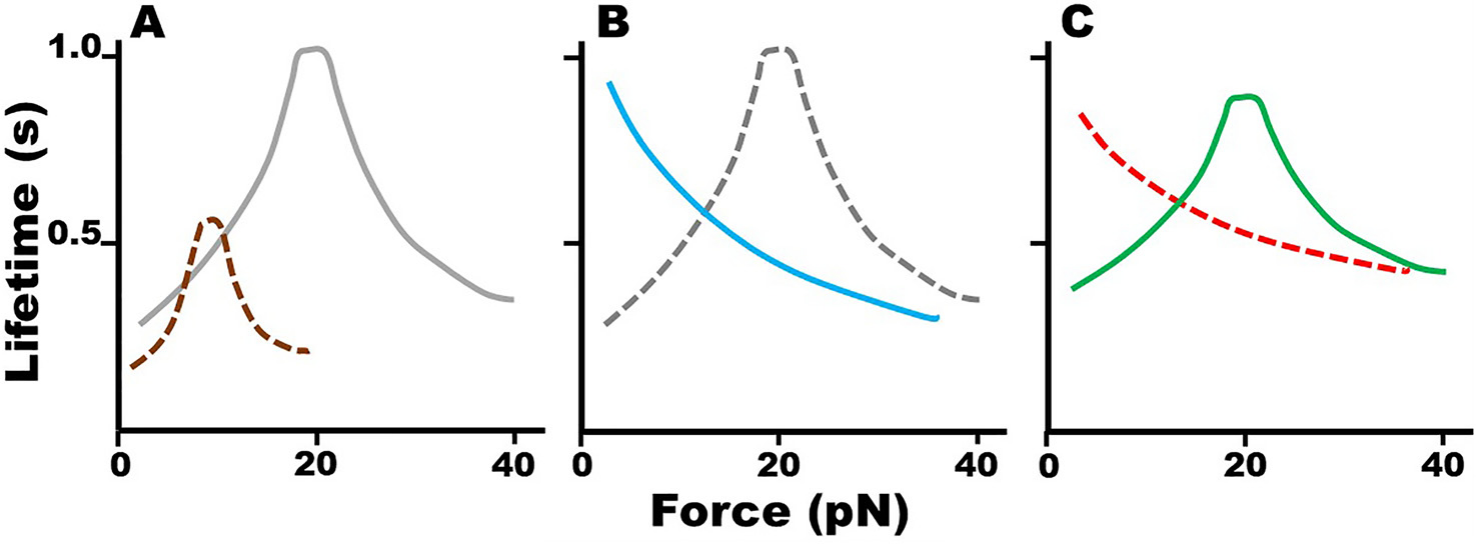
Schematic illustration of actin catch-slip bonds and their regulation by a RhoA–formin module. (a) Bond lifetimes of G-actin–G-actin (brown dashed curve) and G-actin–F-actin (gray solid curve) interactions exhibit a biphasic catch-slip force dependence, with a “catch” region characterized by an increasing lifetime as force increases. Note that the lifetime vs force curve of the G-actin–F-actin bond exhibits a rightward and upward shift relative to that of the G-actin–G-actin bond, doubling the optimal force where lifetime peaks and doubling the peak lifetime. (b) G-actin–F-actin catch-slip bond (gray dashed curve) is switched to slip-only bond by adding mDia1 C-t into the assay system (blue solid curve). (c) Simultaneous treatments of mDia1 N-t inhibit the mDia1 C-t induced conversion of G-actin–F-actin catch-slip bonds to slip-only bonds, restoring the catch-slip phenotype (green solid curve). The inhibitory effect of mDia1 N-t on mDia1 C-t is relieved by RhoA (red dashed curve). Drawn to depict the data from Refs. 28 and 29.

To test the hypothesis that force modulates the dissociation kinetics of actin subunits, Lee used atomic force microscopy (AFM) to measure the lifetime of single bonds between two G-actin molecules and between a G-actin molecule and an F-actin filament. Remarkably, we found that G-actin forms catch bonds with both G-actin and F-actin at low forces where bond lifetimes increase with increasing force. After reaching their respective lifetime maxima at their respective optimal forces, both G-actin–G-actin and G-actin–F-actin bonds turn into slip bonds where bond lifetimes decrease with a further increase in force [Fig. 2(a)]. Compared to the G-actin–G-actin catch bond, the G-actin–F-actin catch bond is more pronounced, with twice the lifetime and doubling the optimal force where the bond lifetime peaks and the catch bond transitions into the slip bond. Similar catch bonds are formed by the barbed end and pointed end of the F-actin with the G-actin. In addition, we used a combined approach of molecular dynamics simulations, mutagenesis, and AFM measurements to elucidate the structural mechanisms of the actin catch bonds. We found that the force-induced formation of the K113:E195 salt bridge between the two interacting actin monomers is a key contributing noncovalent interaction to catch bonds at the atomic level. Mutations at either the 113 or 195 position or both to eliminate this salt bridge progressively suppressed the G-actin catch bonds with both G-actin and F-actin. On the human actin gene ACTA1, the K113E mutation has been reported to involve in nemaline myopathy,^27^ indicating the potential pathological relevance of actin catch bonds. The difference between the G-actin–G-actin and G-actin–F-actin catch bonds was explained by the ability of G-actin to form both long-pitch and short-pitch dimers with the F-actin end but only one of these dimers with another G-actin. These results thus support our first hypothesis that force modulates actin depolymerization kinetics. We interacted with Professor Chien during the conceptualization of the project, discussed with him our results, and published a paper with him in 2013.^28^

## REGULATION OF ACTIN CATCH-SLIP BONDS WITH A RhoA-FORMIN MODULE

To test the second hypothesis that RhoA and formin regulate the force modulated actin dissociation kinetics, Lee used AFM to analyze the actin dynamic bonds in the presence or absence of two mDia1 constructs and RhoA. We found that the addition to solution of the C-terminal construct (mDia1 C-t, consisting of the FH2 domain and DAD) converted the catch bonds of G-actin with both G-actin and F-actin to slip bonds [Fig. 2(b), showing for the G-actin–F-actin interaction case only]. Adding to solution the N-terminal construct (mDia1 N-t, consisting of DID) in the above experiment to allow the DAD–DID binding to auto-inhibit the FH2 domain rescued the actin catch bonds [Fig. 2(c)]. Interestingly, addition of RhoA relieved the auto-inhibition, and enabled the FH2 domain to switch the catch bonds to slip bonds again [Fig. 2(c), showing for the G-actin–F-actin interaction case only]. Molecular dynamics simulations observed that, when formin bound to actin K118 and E117 residues located at the helical segment extending to K113, force no longer induced the K113:E195 interaction, thereby revealing the structural mechanism for the formin modulation of the actin catch bond. Professor Shu Chien worked with us closely in the data interpretation and writing of the paper, which we published with Professor Chien in 2016.^29^

## CONNECTING THE MECHANOBIOLOGY AT THE MOLECULAR LEVEL TO THAT AT THE CELLULAR LEVEL

**FIG. 3.**
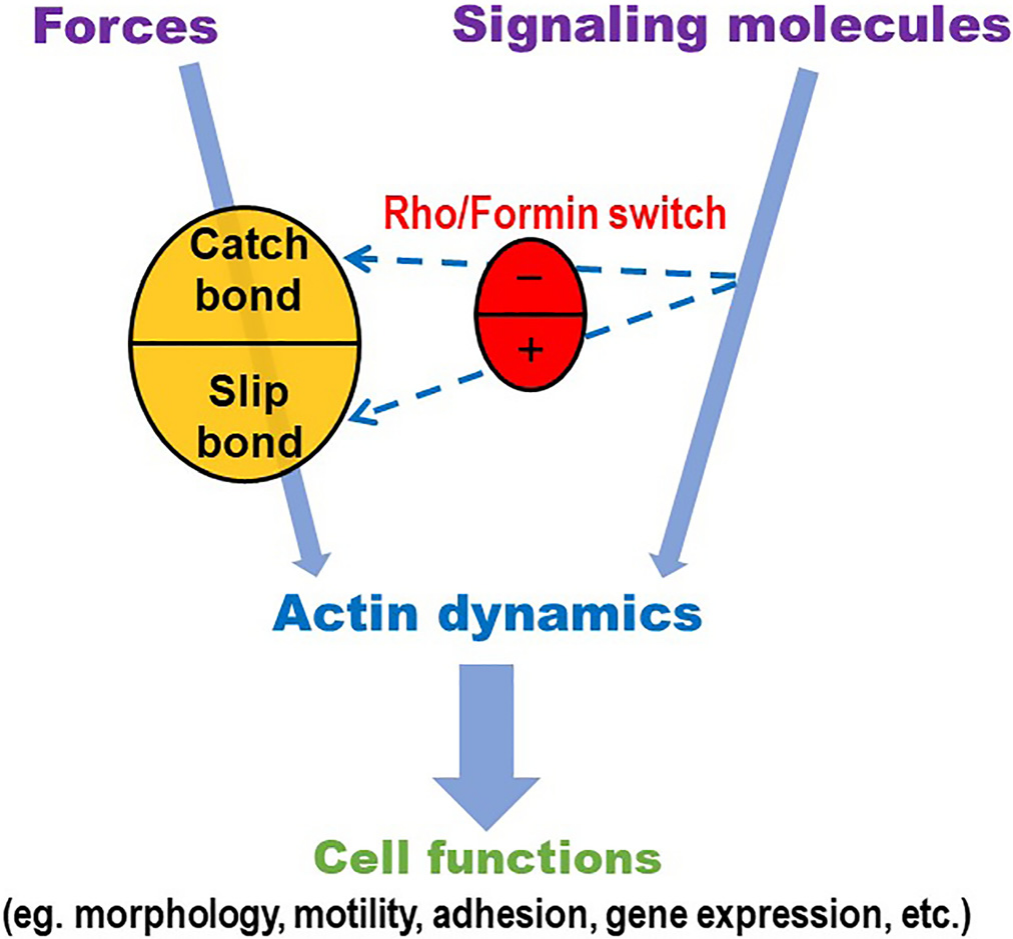
Mechanochemical regulation of actin dynamics. Actin cytoskeleton dynamics is regulated by forces and by biochemical signaling molecules including GTPases. Force modulates actin dynamics by a catch-slip mechanism (gold ovoid) and this biomechanical modulation is regulated by biochemical signaling through Rho and formin (red ovoid and dashed arrows). The Rho–formin module can serve as a switch shifting the force dependence of actin dynamics between catch bonds (with inactivated Rho and formin) and slip bonds (with activated Rho and formin), contributing a crosstalk bridging the dual mechanochemical regulation of actin dynamics, which relays to control various cell functions. Reproduced with permission from Lee *et al.*, Sci. Rep. **6**, 35058 (2016).

The findings that the actin dissociation kinetics exhibits catch-slip bond behavior and that the RhoA–formin module switches actin catch-slip bonds to slip-only bonds have several implications. First, it shows the crosstalk between the biomechanical modulation and biochemical regulation of actin dynamics; such coupling suggests the possibility of synergy and feedback (Fig. 3, dashed arrows). Second, the GTPase-mediated signaling molecules Rho and formin are known to modulate tension-mediated formation and turnover of the actin cytoskeleton in cells (Fig. 3, red ovoid). For this reason, the regulation of actin catch bonds by Rho and formin supports the biological relevance of actin catch bonds (Fig. 3, gold ovoid), although catch bond seems to be an unusual and counter-intuitive biophysical property of actin interaction. Third, the results from our *in vitro* experiment at the molecular level may add to the explanation of the cellular level findings of the Chien lab in 2005.^8^ In the presence of mDia1 and RhoA, actin forms a slip-only bond and the longest bond lifetime occurs at zero force, suggesting that F-actin is most stable in the direction of minimal force. This may favor the directional alignment of actin stress fibers in cells along the direction perpendicular to stretch along which force becomes minimum, providing a possible explanation to the control phenotype in Fig. 1 (top). When mDia1 is inhibited, or when RhoA no longer relieves the auto-inhibition of mDia1, actin forms catch-slip bonds and the longest bond lifetime occurs at the optimal force, suggesting that F-actin is most stable in the direction along which force is at such a level. This may lead to switching in the alignment direction of the actin stress fibers to the direction in parallel to the stretch direction along which force is higher, providing a consistent explanation to the three experimental phenotypes in Fig. 1. Although no technique is available at present to allow the *in vivo* measurement of actin dynamic bonds and their regulation by the Rho–formin module, our speculation suggests a potentially fruitful direction for future studies in the structural and morphological adaptation of cells under dual control of mechanical forces and signaling molecules.

Finally, actin catch bonds may play a role in the mechanosensing of the cell. Much of the published work on mechanobiology (many of which were contributed by Professor Shu Chien) considers the situation where the mechanosensing structures are localized at and/or beneath the cell surface. For example, integrins and their cytoplasmic adaptors talin and vinculin are thought of key elements of a mechanosensing apparatus of many cell types, including vascular endothelial cells.^30^ Like actin, several integrins have been shown to form catch bonds with ligands.^13,14,31–34^ In cells, talin^35^ and vinculin^36^ have been shown to bear forces, which may induce conformational changes in these intracellular proteins.^37,38^ These properties have been suggested to be important to mechanosensing.^30,39–41^ The membrane localization of the mechanosensing structures may explain why the majority of the studies in the field only consider biochemical reaction and diffusion as the main pathway of signal relay from the cell surface to the interior of the nucleus. However, a recent study found evidence that the actin cytoskeleton transmits forces from integrins to the Linker of Nucleoskeleton and Cytoskeleton complex and then through lamina–chromatin interactions to directly stretch chromatin and upregulate DNA transcription to RNA.^42^ The finding of actin catch bonds may broaden the role of actin cytoskeleton in mechanosensing,^16,43^ which had generally been regarded as merely the conduit of transmission from the site of force exertion to the site where force may directly alter structure and function. The broadened view recognizes that such a conduit can be dually regulated biomechanically by force itself and biochemically by the Rho–formin module, which suggests a new form of mechanosensing. This broader view represents a natural progression from cellular mechanobiology, for which Professor Shu Chien has been a thought leader, to molecular mechanobiology, which represents a new direction that Professor Chien helped pave the way.

This new direction includes many frontiers because proteins that bind to the actin cytoskeleton may also bear forces that may also modulate their interactions and conformations, making them potential components of an intracellular mechanotransduction apparatus. These mechanotransductive structures are dynamic, dispersed throughout the cytoplasm rather than concentrated at the cell surface, and are likely coupled to biochemical signaling pathways. The finding of regulation of actin catch bonds by the RhoA–formin module exemplifies such coupling and adds possible regulatory mechanisms to this mechanotransduction apparatus. Our more recent work revealed that at zero force, neither the association kinetics nor the dissociation kinetics of G-actin–G-actin or G-actin–F-actin interactions were affected by formin; the formin effect was only observed when force was exerted on the actin bond.^44^ We also demonstrated a more general mode of force modulation of actin bond stability termed cyclic mechanical reinforcement^45^ where the actin bond lifetime can be drastically prolonged by cyclic forces.^46^ These findings further support the possible mechanotransduction role of the actin cytoskeleton and related proteins, an important new area of molecular mechanobiology that awaits more mechanistic investigations in the future.

## CONCLUDING REMARKS

Ample experimental evidence suggests that mechanical forces modulate the dynamics of the actin cytoskeleton and, in turn, cell functions that are mediated by the tension-induced assembly and stabilization of the actin cytoskeleton. However, in the conventional kinetic analysis of actin polymerization and depolymerization, the kinetics parameters and their regulation by formin are estimated under force-free conditions where soluble proteins are dispersed in the liquid phase.^18,24,26^ Kinetic analyses of actin catch bonds^28,29,44,46^ and actin-myosin catch bonds^47^ expand the conventional analysis from zero force to a range of forces. In general, the use of single-molecule dynamic force spectroscopy to analyze intracellular protein–protein interactions allows one to obtain information on how force modulates these interactions, hence adding an entirely new dimension in the parameter space and opening a new door for mechanobiology studies. Our work was inspired by and in collaboration with Professor Shu Chien, whose attitude toward rigor in scientific research set a role model for us to follow. These studies have connected the mechanobiology of endothelial cells between the molecular and cellular levels, identified an under-studied area, and opened a new avenue for future exploration.

## AUTHOR'S CONTRIBUTION

All authors contributed equally to this work.
